# Unveiling the Potential of Prostate-Specific Membrane Antigen for Precision Diagnosis and Therapy of Prostate Cancer: A Radiopharmaceutical Perspective

**DOI:** 10.21315/mjms2024.31.4.17

**Published:** 2024-08-27

**Authors:** Hishar Hassan, Muhamad Faiz Othman, Zarif Naim Ashhar, Hairil Rashmizal Abdul Razak, Fathinul Fikri Ahmad Saad

**Affiliations:** 1 Centre for Diagnostic Nuclear Imaging, Universiti Putra Malaysia, Selangor, Malaysia; 2 Department of Pharmacy Practice, Faculty of Pharmacy, Universiti Teknologi MARA, Selangor, Malaysia; 3 Department of Nuclear Medicine, Sabah Women and Children Hospital, Sabah, Malaysia; 4 Medical Imaging Program, Department of Health and Care Professions, Faculty of Health and Life Sciences, University of Exeter, Devon, United Kingdom

**Keywords:** prostate cancer, prostate-specific membrane antigen (PSMA), molecular imaging, theranostic, personalised medicine, radiopharmaceuticals

## Abstract

Prostate-specific membrane antigen (PSMA) has proven to be an important target for diagnostic imaging in prostate cancer. As PSMA is overexpressed on the surface of prostate cancer cells, numerous targeted PSMA ligands have been developed. The emergence of PSMA targeting based on small molecules, such as the PSMA-11 ligand (or PSMA-HBED-CC), has led to breakthroughs, such as [^68^Ga]Ga-PSMA-11, for positron emission tomography (PET) imaging of biochemically recurrent or metastatic castration-resistant prostate cancer (mCRPC). In addition, the recent approval of [^177^Lu]Lu-PSMA-617 for the treatment of adult patients with PSMA-positive mCRPC represents an important milestone in prostate cancer therapy. These advances underscore the growing confidence in the use of PSMA-targeted radiopharmaceuticals for the diagnosis and treatment of prostate cancer patients. PSMA-targeted radiopharmaceuticals have been shown to significantly impact treatment planning and clinical decision-making and facilitate the customisation of treatment regimens.

## Introduction

Prostate cancer is the fifth most commonly diagnosed cancer and the seventh leading cause of cancer mortality in Asia (1). Southeast Asian countries, including Malaysia, have more than twice the mortality-to-incidence ratio compared to Japan and South Korea (2). With an ageing population, urbanisation and westernised lifestyles, the incidence of prostate cancer in Asian men is expected to rise rapidly in the near future, including in Malaysia.

Prostate cancer is usually diagnosed by physical examination, biochemical markers, prostate-specific antigen (PSA) testing and conventional imaging techniques, such as magnetic resonance imaging (MRI) and computed tomography (CT), which detect morphological changes in the tissue (3). More recently, the diagnosis of prostate cancer has reached a new level with the use of molecular imaging techniques using positron emission tomography-computed tomography (PET-CT), which can measure the metabolic activity of cells (4). In contrast to conventional imaging techniques, PET-CT offers greater sensitivity and specificity (5).

### The Role of Prostate-Specific Membrane Antigen in Prostate Cancer

Prostate-specific membrane antigen (PSMA) has emerged as the prime target for diagnostic imaging in prostate cancer. It is a type II transmembrane glycoprotein that is overexpressed in prostate cancer, bladder cancer, schwannomas and the tumour neovasculature of many solid tumours (6). In prostate cancer, PSMA is overexpressed 100 to 1,000 times more than in normal cells, making it an interesting target for imaging and therapeutic tools and enabling this ‘image and treat’ strategy to become an important approach for patients with prostate cancer (7).

With the knowledge that PSMA is overexpressed on the surface of prostate cancer, several targeted PSMA ligands have been developed. Small molecule PSMA inhibitors have been introduced to target enzymatic sites. There are three types of motifs for small-molecule inhibitors based on the zinc-binding segments: i) phosphorus-motif, ii) thiol-motif and iii) urea motif (Glu-urea-Glu or Glu-urea-Lys) (8). The phosphorus and thiol motifs bind to the two zinc ions in the active domain of PSMA and the difference between them depends on polarity (8). The urea motif is somewhat unique in that it is internalised into the cell after binding to the active domain of PSMA, making it a preferred analogue that can be labelled with various radionuclides for clinical use (6, 8). Therefore, this brief review focuses on PSMA-urea motifs.

### Development of a Prostate-Specific Membrane Antigen-Targeting Agent

**Figure 1:**
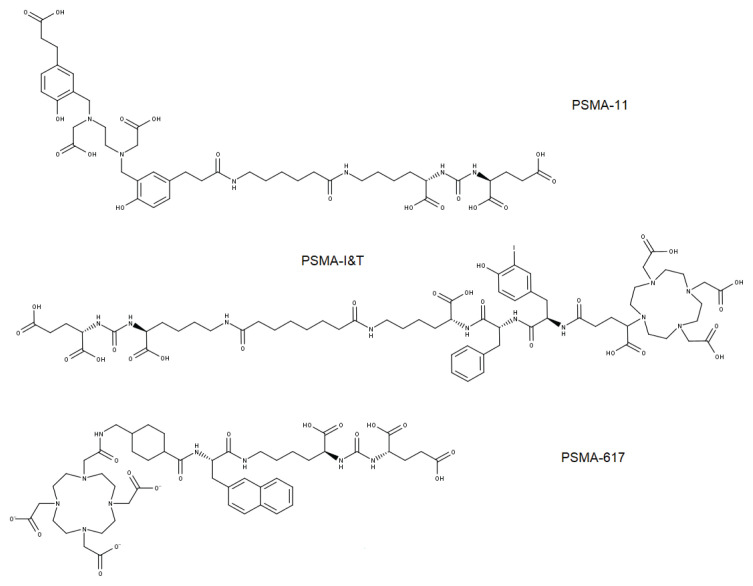
Molecular structures of PSMA analogues based on the Glu-urea-Lys motif

The development of a small molecule PSMA antagonist has a long history, dating back to 2002. However, the breakthrough of the small molecule targeting PSMA came only with the introduction of the PSMA-11 ligand, also known as PSMA-HBED-CC (Figure 1) and radiolabelled with ^68^Galium (^68^Ga) in 2012 (9). This radiopharmaceutical consists of three components: i) a PSMA-targeted urea motif, Glu-urea-Lys, combined with ii) the HBED-CC chelator (N,N’-bis [2-hydroxy-5-(carboxyethyl)benzyl] ethylenediamine) and the DOTA chelator (1,4,7,10-tetraazcyclododecane-N,N’,N”,N”’-tetraacetic acid) via, and iii) an aminocaproic acid linker. The use of HBED-CC and the DOTA chelator make it an ideal candidate for ^68^Ga labelling. Nevertheless, tumour uptake in biodistribution when first introduced was only 3.78% ID/g 30 min after injection of [^68^Ga]Ga-PSMA-11. Later, Weineisen et al. (10) further improved the PSMA ligand using a DOTAGA chelator coupled with DOTA, which became known as PSMA I&T (Figure 1). This resulted in an increase in tumour biodistribution to 4.95% ID/g as [^68^Ga]Ga-PSMA I&T and 7.96% ID/g as [^177^Lu]Lu-PSMA I&T 1 h after injection.

Although HBED-CC has been shown to be an ideal chelator for ^68^Ga labelling, it does not form stable complexes when therapeutic metal radionuclides such as ^177^Lu, ^225^Ac and ^213^Bi are involved (9). To further facilitate labelling with both diagnostic and therapeutic radionuclides, PSMA-617 (Figure 1) containing a urea-binding motif (Glu-urea-Lys) attached to a DOTA chelator was introduced. Further improvement of this ligand resulted in a tumour uptake value of 8.47% ID/g for [^68^Ga]Ga-PSMA-617 and 11.2% ID/g for [^177^Lu]Lu-PSMA-617 (8). It was reported that the PSMA-617 ligand in the form of [^177^Lu]Lu-PSMA-617 has a stronger PSMA-binding affinity, is more internalised into the target organ, has a higher tumour-to-background ratio at late time points and is more rapidly excreted via the kidneys than [^68^Ga]Ga-PSMA-11, marking its significant advantage. In addition, the ligand was reported to form stable complexes not only with ^177^Lu but also with ^64^Cu (9). On 1 December 2020, the United States Food and Drug Administration (US-FDA) approved the use of [^68^Ga]Ga-PSMA-11 for PET imaging of biochemically recurrent or metastatic castrate-resistant prostate cancer (mCRPC) (9), and more recently, on 23 Mac 2022, the US-FDA approved [^177^Lu]Lu-PSMA-617, under the registered name ‘Pluvicto’, for the treatment of adult patients with PSMA-positive mCRPC who were treated with androgen receptor pathway inhibition and taxane-based chemotherapy (11).

### Potential Benefit, Impact of PSMA-Targeted Radiopharmaceuticals on Management of Prostate Cancer Patients and Reported Pitfalls

The use of [^68^Ga]Ga-PSMA-11 in PET imaging has shown promising results in the early detection of prostate cancer, even in patients with a low PSA (7). [^68^Ga]Ga-PSMA-11 was more sensitive and specific than the previously approved PET radiopharmaceutical tracer for prostate, [^11^C]choline. In contrast to PSMA, [^11^C]choline recognises prostate tumour cells through overexpression of choline kinase, resulting in higher endogenous production of choline and greater intracellular uptake by prostate tumour cells (7). In a study by Farsad et al. (12), [^11^C]choline was found to have a sensitivity, specificity, negative predictive value (NPV) and positive predictive value (PPV) of 66%, 81%, 87% and 55%, respectively. Farsad et al. (12) concluded that although [^11^C]choline PET-CT can detect a large number of malignant lesions, there are still a considerable number of false-negative results due to undifferentiated uptake in benign prostate disease (12). In contrast, [^68^Ga]Ga-PSMA-11 PET has a sensitivity, specificity, NPV and PPV of 76.6%, 100%, 91.4% and 100%, respectively (13).

These numbers give confidence in the use of PSMA-targeted radiopharmaceuticals in the diagnosis and treatment of prostate cancer patients. PSMA-targeted radiopharmaceuticals have been reported to greatly influence treatment planning and clinical decision-making, leading to treatment regimen adjustment in 27%–77% of patients (6). The most commonly used pair in the context of radiotheranostics is [^68^Ga]Ga-PSMA-11 and [^177^Lu]Lu-PSMA-617 (6). [^68^Ga]Ga-PSMA-11 plays an important role in predicting and monitoring the response to treatment with [^177^Lu]Lu-PSMA-617 (6). Although the use of PSMA-targeted radiopharmaceuticals in PET-CT has improved the management of prostate cancer patients, it also has its pitfalls for clinical use. In a report by Maurer et al. (14), false-positive findings were found in up to 10% of patients, the causes of which were either non-specific or unclear.

## Conclusion

The rapid development of PSMA-targeted radiopharmaceuticals in imaging and therapy has significantly changed the treatment landscape for prostate cancer patients. Researchers, drug regulatory agencies and the pharmaceutical industry should work hand in hand to ensure that the benefits of these PSMA-targeted radiopharmaceuticals are available to others at an affordable cost.
